# Molecular Recognition and Advances in Antibody Design and Antigenic Peptide Targeting

**DOI:** 10.3390/ijms21041405

**Published:** 2020-02-19

**Authors:** Gunnar Houen, Nicole Trier

**Affiliations:** Department of Neurology, Rigshospitalet Glostrup, Nordre Ringvej 57, 2600 Glostrup, Denmark; gunnar.houen@regionh.dk

Molecular recognition, the specific interaction between molecules by a combination of physical forces, has been a subject of scientific investigation for decades. The physical forces involve a combination of dipole-dipole interactions (van der Waals forces), hydrogen bonds and ionic interactions, and it is the optimal spatial combination of these interactions, that defines the specificity, i.e., the strength of the interaction, measured as an affinity constant, defined by the association and dissociation rate constants: K_a_ = k_on_/k_off_ [1,2,3].

Specific interactions in living organisms are numerous, ranging from base pairing in DNA and RNA, protein folding, protein interactions and many more, constituting the basis of life [4,5,6].

Molecular recognition of foreign substances (self/non-self recognition) is the basis of immune defense against pathogens, spanning from less specific (promiscuous) MHC-peptide interactions to highly specific T cell (antigen) receptor (TCR) recognition of MHC-peptide complexes and from less specific IgM-antigen interactions to highly specific IgG-antigen interactions [7,8,9].

Through the study of the aforementioned specific interactions, scientists have learned to use natural molecules as reagents and have developed new reagents based on the same principles and physical forces.

This issue of IJMS, entitled “Advances in Antibody Design and Antigenic Peptide Targeting” aims to give a status of the current “state-of-the-art” in specific molecular recognition. The issue contains articles on molecular recognition in antigen-antibody complexes and the production and use of antibodies, recombinant or vaccine-induced, as therapeutic agents [10,11,12,13,14,15,16,17,18].

Nature’s own amino acid-based reagents, peptides and antibodies, are cornerstone reagents in molecular biology, but have been successfully combined in the development of peptide antibodies, one of the most successful classes of molecular recognition molecules [17]. Similarly, nucleic acid-based reagents have not only been invaluable in molecular biology in the form of oligonucleotides, e.g., when used for polymerase chain reactions (PCRs), but have also begun to be used as specific recognition molecules in the form of aptamers, self-folding three-dimensional polynucleotides, which can be selectively amplified from libraries by PCR [17].

In recent years, designed antibody-like molecules and nucleic acid-based recognition molecules have been intensely studied, but there is still a long way to go before these reagents can effectively rival nature’s own reagents, peptides and antibodies. 
